# A Gaze-Driven Evolutionary Algorithm to Study Aesthetic Evaluation of Visual Symmetry

**DOI:** 10.1177/2041669516637432

**Published:** 2016-03-22

**Authors:** Alexis D. J. Makin, Marco Bertamini, Andrew Jones, Tim Holmes, Johannes M. Zanker

**Affiliations:** Psychological Sciences, University of Liverpool, Liverpool, UK; Acuity Intelligence Ltd, Reading, UK; Royal Holloway, University of London, Egham, UK

**Keywords:** Symmetry, aesthetics, preference, eye tracking, evolutionary algorithm

## Abstract

Empirical work has shown that people like visual symmetry. We used a gaze-driven evolutionary algorithm technique to answer three questions about symmetry preference. First, do people automatically evaluate symmetry without explicit instruction? Second, is perfect symmetry the best stimulus, or do people prefer a degree of imperfection? Third, does initial preference for symmetry diminish after familiarity sets in? Stimuli were generated as phenotypes from an algorithmic genotype, with genes for symmetry (coded as deviation from a symmetrical template, deviation–symmetry, DS gene) and orientation (0° to 90°, orientation, ORI gene). An eye tracker identified phenotypes that were good at attracting and retaining the gaze of the observer. Resulting fitness scores determined the genotypes that passed to the next generation. We recorded changes to the distribution of DS and ORI genes over 20 generations. When participants looked for symmetry, there was an increase in high-symmetry genes. When participants looked for the patterns they preferred, there was a smaller increase in symmetry, indicating that people tolerated some imperfection. Conversely, there was no increase in symmetry during free viewing, and no effect of familiarity or orientation. This work demonstrates the viability of the evolutionary algorithm approach as a quantitative measure of aesthetic preference.

## Introduction

The scientific study of human aesthetic preference has a long history, beginning with early observations by Fechner (1876). Recently, there has been a resurgence of interest in this area, with new conferences, journals, and the application of modern techniques from cognitive neuroscience (Chatterjee, 2011). We are not at the stage where empirical aesthetics can provide a workable recipe for the production of appealing designs. However, theories inspired by cognitive science have led to insights into the nature of preference formation and the appreciation of art (Arnheim, 1974; Leder, Belke, Oeberst, & Augustin, 2004; Ramachandran & Hirstein, 1999; Reber, 2012; Van de Cruys & Wagemans, 2011; Zeki, 1999). Moreover, empirical studies have also provided reliable results: For example, symmetrical abstract patterns are rated as more beautiful than random patterns by the majority of people (Eisenman, 1967; Eysenk, 1941; Jacobsen & Höfel, 2002).

The appeal of symmetry is interesting because it is not limited to humans and is thus unlikely to be a cultural whim. Many species have a preference for symmetrical mates (Møller & Thornhill, 1998) and symmetrical foods (Wignall, Heiling, Cheng, & Herberstein, 2006). Symmetry might be a predictor of attractiveness ratings in faces (Rhodes, Proffitt, Grady, & Sumich, 1998) and bodies (Bertamini, Byrne, & Bennett, 2013; Tovee, Tasker, & Benson, 2000), while Ramachandran and Hirstein (1999) listed preference for symmetry as one of their laws of artistic experience. There are plausible arguments for why symmetry should be so appealing: The body plan of most animals has bilateral reflectional symmetry, so if development proceeds according to the genetic template, adult phenotype should be approximately symmetrical. Symmetry thus indicates health and reproductive fitness (Grammer, Fink, Møller, & Thornhill, 2003). Prevalence of symmetry in human designs and abstract patterns could be an overgeneralization of a perceptual mechanism designed to detect health and genetic quality.

On the other hand, attractiveness of symmetry is not some fixed rule in nature. The evidence that people find symmetrical faces and bodies most sexually attractive is mixed: The effect size could even be zero after correcting for publication bias (van Dongen, 2011). The correlation between body symmetry and developmental integrity is also weak (e.g., Hope, Bates, Dykiert, Der, & Deary, 2015). Moreover, the size of the asymmetries in animals is small compared with the size of the features themselves. Given lab work on symmetry detection, it seems that the putative “fitness signal” might often be invisible. This is especially important given additional degradation of visual signals in natural settings, where viewing conditions are disoptimal (Swaddle, 1999). It has also been noted that symmetry is confounded with averageness in a population: That is, *symmetrical animals are average animals*. The significance of this was highlighted by Enquist and Johnstone (1997), who presented an artificial neural network with two sets of training stimuli. The training patterns were symmetrical on average, but individually, they were either lopsided to the left or right. If the training sets were similar, then symmetrical test stimuli produced a larger response than any of the lopsided training patterns alone. Thus, increasing sensitivity to symmetrical variants emerges whenever networks exhibit generalization, and when they are exposed to a fluctuating population with a symmetrical mean. Both these conditions are met in nature, and the emergent strong visual response to symmetry could result in positive evaluation. A related view is that the visual system is tuned to symmetry as part of the process of 3D shape analysis, and preference for symmetry may be a by-product of efficient processing (Mascalzoni, Osorio, Regolin, & Vallortigara, 2012).

Despite the well-rehearsed debate about the evolution of symmetry preferences, the conditions under which this positive evaluation occurs are yet to be fully documented. When participants are forced to rate the aesthetic appeal of abstract patterns, symmetry is usually liked: But is symmetry evaluated spontaneously, even when people are not forced to make an aesthetic evaluation? Jacobsen and Höfel (2003) addressed this question by recording event-related potentials (ERPs) produced by subjectively beautiful and ugly abstract black and white patterns. ERPs sensitive to regularity were found in all conditions, while ERPs that distinguished between beautiful and ugly patterns were only present during explicit evaluation. Höfel and Jacobsen (2007) found comparable results and concluded that: “Aesthetic appreciation of beauty appears to require intention and is not spontaneous in character” (p. 30).

To reiterate, ERP results show that the visual system automatically discriminates symmetrical and random patterns, but that affective systems *do not* spontaneously *evaluate* the patterns. There is a posterior ERP component that differs for symmetrical and random presentations. This is found when people are attending to regularity and classifying the patterns as regular or random, and when they are attending to some other feature of the display, such as the color of the elements (Makin, Rampone, & Bertamini, 2015). This automatic visual processing is apparently in stark contrast with aesthetic evaluation: ERPs that distinguish between subjectively beautiful and ugly patterns are not generated when unless people are engaged in aesthetic evaluation (Jacobsen & Hofel, 2003; Höfel & Jacobsen, 2007). When we talk about “evaluation” of symmetry, we are *not* talking about visual detection and processing of symmetry (which is automatic), but rather aesthetic evaluation, that is, is the pattern good or bad, attractive or unattractive, pleasing or displeasing, and so on (which may or may not be automatic).

More recent research has employed implicit measures of preference to ascertain the conditions for automatic evaluation. Studies using the Implicit Association Test (IAT; Nosek, Greenwald, & Banaji, 2007) have shown that symmetry is more easily associated with positive words and random with negative words (Bertamini, Makin, & Rampone, 2013; Makin, Pecchinenda, & Bertamini, 2012). However, the IAT procedure merely shows that the conceptual dimensions “symmetry-random” and “positive-negative” are orientated in a common direction (see Walker, 2012 for other examples of dimensional correspondences). The IAT experiments do not demonstrate spontaneous aesthetic evaluation *in a strong sense*. Other work has used the affective priming technique (Bertamini, Makin, & Pecchinenda, 2013). Abstract patterns were briefly flashed, followed by a word, which participants classified as positive or negative. If symmetrical patterns are spontaneously evaluated as positive, they should facilitate processing of positive words. Likewise, if random patterns are spontaneously evaluated as negative, they should facilitate processing of negative words. However, no such priming effect was found without modification of the procedure that forced the participants to classify the prime pattern as well as the words. In summary, preference for symmetry over random is nearly universal when people explicitly consider aesthetic merit, but there is no evidence that the irresistible attractiveness of abstract symmetry automatically *pops out* and forces itself on the observer. The first question in the current work is whether there are conditions in which symmetry is evaluated automatically.

Symmetry can be detected when it is less than perfect, for example, when the individual elements are jittered around a symmetrical template (Barlow & Reeves, 1979). Do people like perfect symmetry in abstract patterns most? Different models of aesthetics make different predictions: Zeki (1999) argues that artists are experts in isolating and maximizing excitation of visual modules. It is known that perfect symmetry produces more neural excitation in visual areas than degraded symmetry (Sasaki, Vanduffel, Knutsen, Tyler, & Tootell, 2005). According to the optimal stimulation account of Zeki (1999), perfect symmetry should be preferred over noisy symmetry. The fluency-attribution account of aesthetics suggests people are sensitive to the efficiency of their own perceptual and cognitive processes and that fluent processing has positive hedonic tone, which can sometimes lead to a positive evaluation of the stimulus (Reber, 2012). Again, perfect symmetry is detected more quickly, so should theoretically be preferred. Others have considered the importance of perceptual ambiguity in aesthetic experience; for example, van de Cruys and Wagemans (2011) considered appreciation of art within the framework of the hierarchical predictive coding model (Clark, 2013; Rao & Ballard, 1999). They argue that the essence of aesthetic appeal and the fascination of art arise from resolution of perceptual ambiguity, resulting in a sudden reduction of prediction error signals. This account suggests that hidden regularity with added noise may be more appealing than a relatively stable and predictable perfect regularity. More generally, the idea that perfect symmetry is too rigid and unlifelike has been traced as far back as Kant (as discussed in McManus, 2005). Therefore, the second question in the current work is whether people prefer perfect or imperfect symmetry.

A third question about symmetry preference concerns its development over time. People’s preferences do not remain stable and fixed indefinitely. Indeed, the dynamics of prototype formation and challenge in human creations have been well studied, and liking for a particular design or artistic genre cannot be isolated from history (Cutting, 2003). For example, preference for car design is fundamentally shaped what has gone before, and what seems modern or innovative (Carbon, 2010). Meanwhile, it has long been known that mere exposure to stimulus can increase its appeal (Bornstein, 1989; Zajonc, 1968). So, even though preference for symmetry is possibly the result of evolutionary processes and not relatively short-term cultural dynamics, there could nevertheless be some moderating influence of familiarity and exposure (Tinio & Leder, 2009).

In summary, the literature about the aesthetic appeal of symmetry leaves three unresolved questions: (a) Is symmetry evaluated spontaneously? (b) Do people prefer perfect symmetry or ambiguous symmetry? (c) Do preferences for abstract patterns systematically shift with familiarity? We address these three research questions using a *gaze-driven evolutionary algorithm technique* (GDEA), which has already been used in other contexts, for example, to study preference for color–shape combinations and commercial graphics (Holmes & Zanker, 2008a, 2008b; Holmes & Zanker, 2012, 2013).

**Figure 1.**
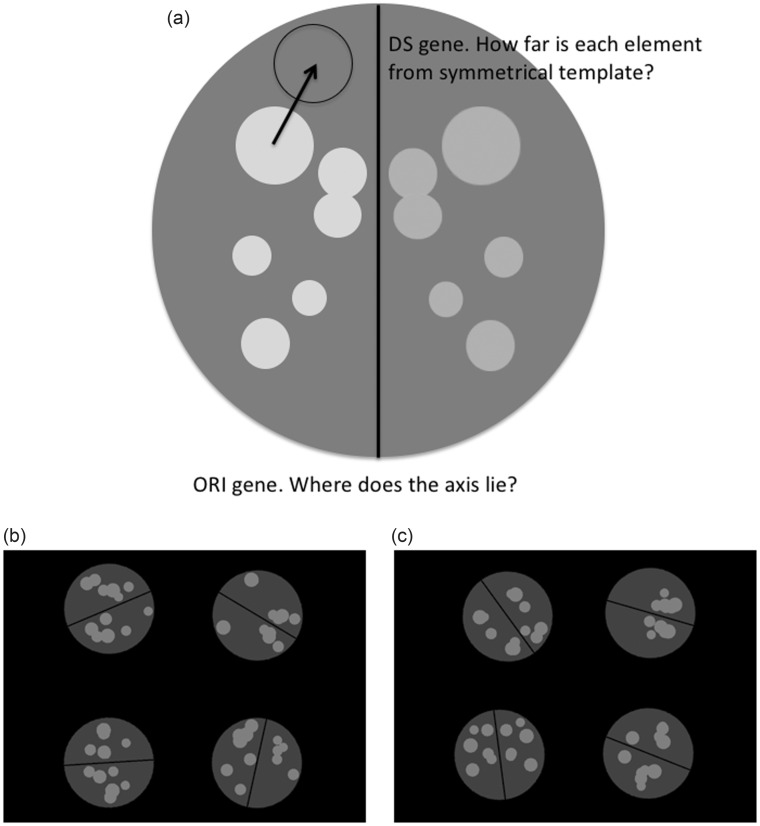
(a) Construction of a single phenotype from the distance from symmetry (DS) and orientation (ORI) genes. This is a schematic only, not an accurate rendering of the stimuli, like those shown in Figure 1(b) and (c). The dots were all the same gray color in the real stimuli. (b) Screen shot from the first generation without systematic genetic drift. (c) Screenshot from a later population typical of the preference group, where the participants had been fixating the more symmetrical patterns.

In our study, the stimuli were 12-dot computer-generated patterns, with an orientation and a degree of symmetry (Figure 1). The essential point of the genetic algorithm is the visible characteristics of the stimuli are like the phenotype, controlled by a virtual genotype. Real-time feedback from an eye tracker is used to assess which phenotypes were most successful in attracting the participant’s gaze. Over the course of the experiment, the stimuli evolve to become more and more like those stimuli that were frequently fixated in earlier generations. The population of virtual genes at the end of the experiment therefore reflect the combination of characteristics that best attracted participants gaze and were fixated longest. We compared the trajectory of virtual genetic drift in three different groups of 18 participants: (a) Participants explicitly looking for symmetry, (b) participants explicitly looking for their preferred patterns, and (c) participants in a free-viewing condition, without explicit instructions.

There are two advantages to using feedback from an eye tracker, rather than a mouse or touchpad. First, it allowed us to explore preference in the free-viewing condition. Second, it allowed us to efficiently obtain a continuous measure of fitness for each of the four patterns presented, based on carefully calibrated oculomotor metrics, rather than a single binary choice.

## Method

### Participants

Fifty-four participants were involved (aged 17 to 47, 17 men, 4 left handed). Participants received course credit or £5 reimbursement for travel expenses. The study had local ethics committee approval and was conducted in accordance with the Declaration of Helsinki (Revised 2008).

### Apparatus

Participants sat 57 cm from a 1024 × 768 resolution 75 Hz LCD monitor. The screen width was 34° of visual angle. Head position was stabilized with a chin rest. The position of the right eye was monitored continuously with an Applied Science Laboratories Eye Trac-6000 infrared eye tracker (ASL, Bedford, MA) sampling at 120 Hz. The GDEA was implemented in Matlab 2011a (Mathworks, Cambridge, MA) using code similar to previous work (Holmes & Zanker, 2013). Stimuli were rendered using functions from the *Cogent* library (www.vislab.ucl.ac.uk/cogent.php). To this end, fixations were assessed to assign fitness scores to the genotypes.

### Stimuli and Procedure

Each trial began with a 1 s fixation period with a central cross, followed by an array of four dot patterns, visible for approximately 5 s. Generation steps and example patterns are shown in Figure 1. All patterns were presented on a circular background with a diameter, which was set a quarter of screen width (8.5°). The background was black (RGB = 0, 0, 0), and the circular background was dark gray (RGB = 0.25, 0.25, 0.25). The patterns were made of 12 dot elements, which were mid-gray (RGB = 0.5, 0.5, 0.5). Dot size was chosen at six incremental steps between 0.9° and 1.5°. A single dot of each size was presented on each side of a visible black midline. Previous work has show that vertical reflection is most salient, followed by horizontal then oblique, but that that these effects disappear if orientation is predictable (Wenderoth, 1994). We wanted people to see the degraded symmetry even for the difficult orientations, despite the fact that orientation was not predictable. We thus included a visible midline to make the axis reflectional symmetry and the orientation as obvious as possible. Dots were free to overlap. The four stimuli were not symmetrically placed in the four quadrants of the screen, but a degree of jitter was introduced on X and Y dimensions (minimum 0°, maximum 0.33°). This feature was designed to prevent the four stimuli forming a large perceptual grouping that was itself symmetrical.

### Genetic Algorithm

The genetic algorithm has, at its core, a simulation of the processes of natural selection that have shaped living organisms. The stimuli seen on the screen are *phenotypes*, and their features are coded by a virtual *genotype*. In our case, the virtual *chromosome* has a symmetry gene (coding deviation from perfect symmetry as a distance, *DS gene*) and an orientation gene (coding angle of the symmetry axis, *ORI gene*). There was also a binary angle gene (*BINA gene*), which allowed for meaningful genotype-to-phenotype translation of the ORI gene, as described later. Other stimulus parameters were chosen independently of the genetic algorithm (e.g., the size of the background disks did not vary, and neither did the color of the stimuli).

#### Three stimulus genes

The DS gene could vary continuously between 0° and 4.25°. This range created phenotypes which were either totally symmetrical or where the symmetrical template was completely invisible (the upper limit was somewhat arbitrary, but allowed for complete obliteration of symmetry in the visible phenotype). For each pattern, position was chosen randomly for half the elements, and the mirror image of these positions were set for the other half. This meant that it was highly unlikely that two patterns were identical, even if they had the same DS alleles. The DS gene coded the distance a dot deviated from this symmetrical template. The direction of deviation was randomized. For a value of 0, the pattern would be perfectly symmetrical, while larger values for the DS gene code for larger deviations from symmetry. If the combination of element position and DS gene resulted in element coordinates beyond the perimeter of the patterns or midline, new values for the random parameters were chosen until the constraints were satisfied.

The ORI gene coded the orientation of the axis of symmetry. This could vary between 0 (vertical) and 90 (horizontal). A third gene-coded angle (BINA gene). This gene was binary—that is, either 1 or−1, and ORI × BINA set the actual orientation of the axis. This implementation detail is important because it meant the ORI gene could meaningfully be interpreted during analysis: Consider the consequences of an ORI gene that could vary between 0 and 360. The alleles 0 and 360 would produce perceptually identical results, but be as different as it is possible to be on the scale. Likewise, if the ORI gene could range from 0 to 180, the same would be true for horizontal orientations—The extreme values for the gene would lead to perceptually identical horizontal symmetry. Having a range of 0 to 90 allows a meaningful match between genotype and phenotype, which is essential for assigning fitness scores based on oculomotor feedback. The BINA gene performs a secondary function of allowing leftward and rightward tilts within these constraints, and was not analyzed.

#### Simulated genetic evolution over generations

Some parameters of the genetic algorithm had to be selected in advance: for example, the number of generations, trials within a generation, and so on. It is not clear a priori what the optimal parameters should be. However, in making these decisions, we drew on pilot data, where we were able to drive evolution deliberately in a particular direction at will, say toward symmetry or toward vertical patterns. We also drew on previous experience with oculomotor genetic algorithms and with these codes (Holmes & Zanker, 2012), which suggests that the concurrent presentation of four stimuli is optimal for evaluating oculomotor fitness in such an evaluation task (Holmes, 2010).

Each participant starts with a randomly generated population of 48 different patterns (phenotypes) which were generated from the specific range of possible values (alleles) for the DS, ORI, and BINA genes. Adopting the methodology outlined in Holmes & Zanker (2012) and established in Holmes (2010), a subset of 16 population members were presented for fitness evaluation based on eye movements, with fitness for the remaining 75% of the population being estimated based on Euclidian distance of the genetic representation of the phenotype (chromosome) from the presented sample phenotypes. This method reduces the elapsed time of the experiment, and consequently the risk of fatigue effects and perceptual learning. Figure 1(b) shows a screenshot from the first generation, where the four phenotypes are set by four genotypes from starting population.

Each run of the experiment comprised 20 generations, which is sufficient for reliable convergence with such a small number of genes (Holmes, 2010). While participants were viewing the stimuli, the fixations were recorded continuously with an eye tracker. After presentation, each phenotype was assigned a *fitness score*, based on the amount of time the pattern was fixated by the participant (and other oculomotor metrics, see subsequent text). The fitness scores of the presented phenotypes were then used to estimate fitness scores for the (32) unpresented population members. The scored population members were then entered into a series of tournaments consisting of random samples of Size 3, with the two fittest members from each sample being used as “parents” in a reproduction process which combined genes from both members together with a random mutation process to produce new offsprings. Thirty-two such offsprings were created and used to replace the least-fit 32 members of the population, thus the population size remained constant throughout, and this process has been shown to ensure that the average fitness of the population increases monotonically with each generation (see Holmes, 2010 and Holmes & Zanker, 2012 for details).

If participants had no systematic tendency to fixate particular phenotypes, the final population would not differ from the first population and would have approximately the same genetic makeup. However, if participants systematically fixated certain phenotypes (e.g., those with near-perfect symmetry), the population will evolve to be more like the fixated phenotypes (e.g., more and more symmetrical).

All participants viewed two evolutionary runs which had exactly the same settings and parameters but were totally independent of each other. For half the participants, the runs were interleaved. For the other half, the runs were separate blocks. In supplementary analysis, we found no interactions involving the between subjects factors Run (One, Two) or Run Arrangement (interleaved runs, separate runs) and the within subjects factor Generation (1 to 20). This was true for both the DS gene, max *F* (10.86, 521.39) = 1.134, *p* = 0.332, and the ORI gene, max *F* (5.78, 555.30) = 1.468, *p* = 0.189. We therefore averaged data from both runs for all subsequent analysis.

#### Assigning fitness scores based on oculomotor statistics

In order to simulate natural selection, there needs to be some means to calculate, or at least estimate, the fitness of each population member. This fitness score is used to select parents for future offspring using a tournament-based method that ensures that the strongest population members are most likely to pass their features on to the next generation (Miller & Goldberg, 1995). Here, a combination of oculomotor measures associated with aesthetic preference was taken from the eye-tracker recordings. This measure uses a weighted sum of the total time spent fixating each image. To quantify this, a dispersion fixation filter was used (I-DT; Salvucci & Goldberg, 2000), with a gaze position radius of 0.5° and a minimum duration of 100 ms. The sequence of first fixations on each image and the amount of revisits to each image was used to calculate a score between 0 and 1. The fitness score represents the amount of attention being directed toward each image. The speed of oculomotor responses was not taken into account when calculating fitness. Because only 25% of the population is presented in each generation, the fitness scores for the remaining 75% of the population were estimated using a least-squares approximation of the fitness based on the scores for the eye-tracked phenotypes and their proximity to the unpresented (non–eye-tracked) phenotypes. Further mathematical details are provided in Holmes and Zanker (2012).

### Symmetry, Preference, and Free Viewing Tasks

A crucial feature of our study was task instruction, which varied between groups of participants. Eighteen participants were given the instruction to look for the most symmetrical pattern of the four available, and fixate it until the end of the 5 second trial (symmetry instruction). This should provide the strongest possible selection pressure toward low DS alleles, and very symmetrical phenotypes. This provided a baseline: How symmetrical would the final population be if symmetry were the only criterion for fixation?

Another 18 participants were told to look for the patterns they liked most, with no further instruction (preference instruction). Here, we can see whether symmetry evolves in the population and to what degree (perfect or less than perfect). We can also see if preferences change over time. For example, participants may begin to like the less regular patterns once symmetry becomes too familiar, resulting in a subsequent rebound toward random in later generations. Finally, 18 participants were not given specific task instructions, other than to pay close attention to the stimuli on the screen, rather than looking somewhere else in the room (free-viewing instruction). If symmetry is attractive in the absence of any instructions, the population should evolve to become more symmetrical in this group as well. After all, it is thought that people spontaneously seek out and attend to aesthetically pleasing stimuli (Biederman & Vessel, 2006). We also ran the program without a participant or any feedback from the eye tracker 18 times. This ensured that apparent effects were due to gaze contingent evolution, and not some unforeseen artifact in the program (control condition).

## Results

**Figure 2.**
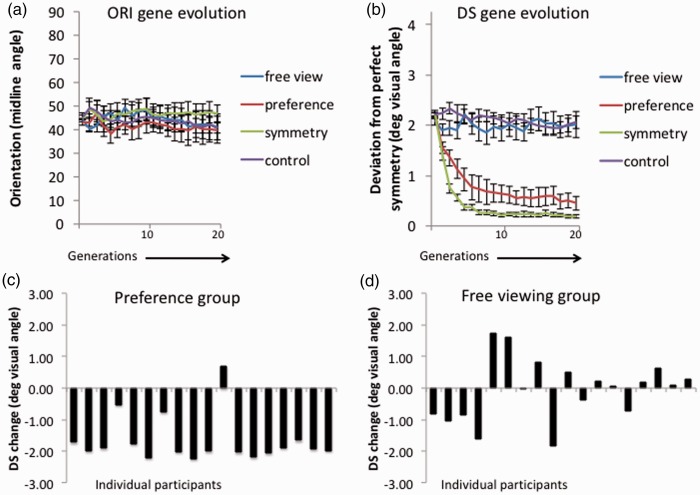
(a) Change in the orientation (ORI) gene (which codes angle of the visible midline) from Generations 1 to 20 (averaged over all participants). (b) Change in the DS gene (which codes deviation from perfect symmetry in degrees of visual angle) from Generations 1 to 20 (averaged over all participants). Note the reduction in population DS in the preference and symmetry groups only. (c) DS change (from Generation 1 to the last 5 generations) in the preference group. (d) DS change in the free-viewing group. In Figure 2(c) and (d), each bar represents an individual participant. Error bars on Figure 2(a) and (b) are ±1 *Standard Error of the Mean*. Note that in the control condition there were no participants, and no feedback from the eye tracker. DS = deviation–symmetry.

For the three groups of participants in the different instruction conditions (symmetry, preference, and free viewing), we measured the *median value* for the DS and ORI genes in Populations 1 to 20. There were no systematic effects on orientation (Figure 2a). Evolution of the DS gene is shown in Figure 2(b). For the 18 free-viewing participants, who were given no instruction, there was little shift away from the start population. This data were very similar to the control condition, where there was no participant and no feedback from the eye tracker. For 18 participants who were looking for symmetry, the population changed to become more and more symmetrical. The most interesting result was from the 18 participants who were looking for their preferred pattern. In this case, there was again a clear shift toward symmetrical populations, as in the symmetry group. However, the DS gene did not shift so far in the preference group as in the symmetry group. This suggests that participants were happy with less-than-perfect symmetry. Figure 1(c) shows screenshot typical of the last generation of the preference task, where DS reach a median value of around 0.5° visual angle.

Statistical analysis confirmed these impressions. Median values for DS and ORI genes in each population were submitted to mixed analyses of variance. There was one within-subjects factor, generation, with 20 levels, and one between-subjects factor, instruction, with 4 levels, instruction (symmetry, preference, free viewing, control). The Greenhouse Geisser correction factor was applied when the assumption of sphericity was violated. For the ORI gene, there were no effects of interactions, max *F* (5.34, 363.29) = 1.153, *p* = .332. For the DS gene, there was a main effect of generation, *F* (4.53, 307.98) = 35.46, *p* < .001, and a generation ×instruction interaction, *F* (13.59, 307.98) = 9.94, *p* < .001, and a main effect of the between-subjects factor, instruction, *F* (3, 68) = 39.00, *p* < .001.

Next, we examined the effect of generation on each Instruction condition separately (symmetry, preference, and free view), and included the control condition data in the analysis for comparison. For the free view and control analysis, there was no effect of generation. *F* (4.10, 139.44) = 0.739, *p* = .561, and no generation × instruction interaction, *F* (4.10, 139.44) = 1.45, *p* = .220. There was no overall difference between free-viewing and control conditions, *F* (1,34) = 0.269, *p* = .607. In short, there is no evidence that the participants engaged in any systematic form of preferential looking in the free-viewing condition at all.

In contrast, for the comparison of symmetry and control conditions, there was a main effect of generation, *F* (3.20, 108.62) = 38.43, *p* < .001, a main effect of instruction, *F* (1,34) =208.73, *p* < .001, and a generation × instruction interaction, *F* (3.20, 108.62) = 24.703, *p* < .001).

A similar pattern of results was found when comparing the preference and control analysis. There was again a main effect of generation, *F* (3.50, 119.08) = 17.41, *p* < .001, a main effect instruction, *F* (1, 34) = 51.84, *p* < .001, of generation × instruction interaction, *F* (3.50, 119.08) = 9.09, *p* < .001). Again, this confirms that the participant’s oculomotor behavior drove evolution of symmetrical phenotypes.

Finally, the most interesting comparison was between the symmetry and preference groups. There was a main effect of generation, *F* (3.88, 131.89) = 82.07, *p* < .001. The DS gene reached lower values in the symmetry group than in the preference group, *F* (1, 34) =5.84, *p* = .021. There was no interaction, *F* (3.88, 131.89) = 1.790, *p* = .137.

Figure 2(b) shows that evolution of the DS gene in the symmetry and preference groups can be broken into two distinct episodes. In Generations 1 to 10, there was a clear reduction in median DS values and a minimum was reached. In the second half, Generations 11 to 20, there was no further genetic changes, and there was no evidence of a rebound-to-random in the preference group. We analyzed these impressions by looking at linear effects in the first and second halves of the experiment. For the earlier generations, there was a significant linear contrast in the symmetry group, *F* (1, 17) = 614.68, *p* < .001, and in the preference group, *F* (1, 17) = 43.44, *p* < .001. For the later generations, evolution of the DS gene reached a lower plateau, and there was no significant linear contrast in the preference group, *F* (1, 17) = 3.97, *p* = .063, or symmetry group, *F* (1, 17) = 2.39, *p* = .140).

Finally, we explored individual differences between participants in the preference and free-viewing groups in more detail. Figure 2(c) shows the difference in DS between the first generation and the average of the final five generations in for each subject in the preference group. All but one subject had a preference for symmetry, leading to negative values for DS change. In the free-viewing condition (Figure 2d), perhaps 6 of the 18 subjects spontaneously engage in similar behavior, resulting in a substantial negative DS change and consequential shift toward symmetrical populations. However, an equal number pushed evolution in the opposite direction, that is, toward random.

## Discussion

When participants were explicitly told to look for the most symmetrical patterns, the final population was far more symmetrical than the first population (Figure 2b). This condition provides a baseline, showing maximum amount of symmetry that can evolve under the parameters of our evolutionary algorithm, when the participant’s only aim was to provide the selection pressure toward increasing symmetry. Here, people were trying to breed symmetrical population of patterns by biasing reproduction in favor of high-symmetry genes. This can be contrasted with the preference condition. Participants were told to look for the patterns they liked most, without any mention of symmetry. They again drove the population toward increasing symmetry. However, this selection pressure was not so strong, and a greater level of noise was tolerated in the later populations.

Preference for symmetrical abstract patterns has been demonstrated with a number of techniques, both implicit and explicit (Bertamini, Makin, & Pecchinenda, 2013; Jacobsen & Höfel, 2002, 2003; Makin et al., 2012). Most theories of visual aesthetics can accommodate preference for symmetry, while preference for reflectional symmetry has been shown in many species, including bees (Rodriguez, Gumbert, de Ibarra, Kunze, & Giurfa, 2004), swallows (Møller, 1992), and humans (Rhodes et al., 1998). Symmetry is a positive feature in mate and food selection, possibly because it reliably signals genetic fitness (Grammer et al., 2003). Infants may already be attracted to symmetrical configurations (Bornstein, Ferdinandsen, & Gross, 1981). Preference for symmetry is not just the most widely replicated finding in scientific aesthetics; it is perhaps the finding that has attracted the most theoretical consideration. This study is consistent with these results but also provides new insights.

In empirical aesthetics, observers often are asked to evaluate a pattern using a Likert scale, or to choose their preferred option from those presented (Palmer, Schloss, & Sammartino, 2013). However, the genetic algorithm approach allows a richer and more ecologically valid measure of preference. The evolution of the DS gene in the preference group reflects the combination of multiple and realistic factors that can be seen as an expression of liking. We can say people spent more time looking at the more symmetrical patterns. More specifically, assigned fitness increased as the result of (a) involuntary saccades and fixations driven by visual salience, (b) explicit choices about which patterns are attractive, and (c) choices about how much to explore the array once a reasonably symmetrical pattern has been foveated. Oculomotor behavior while looking for preferred patterns contains more information than a single rating. Our evolutionary algorithm integrates this information and gives a multifaceted measure of preference. With this new metric, we can see that our participant’s preoccupation with symmetry was somewhat less than maximal during the preference task. People were not simply looking for the most symmetrical pattern and fixating on it until the end of the trial, even though symmetry was clearly a major driving their preferences.

Different theoretical models of preference formation make diverging predictions about the appeal of perfection. Some accounts emphasize optimal stimulation of visual modules (Zeki, 1999) and efficient perceptual processing (Reber, 2012). These accounts predict that people should like perfect symmetry the most. Others suggest that aesthetics appeal requires ambiguity and dynamic resolution of perceptual conflict (Van de Cruys & Wagemans, 2011), so symmetry with some noise may be preferred. The current results suggest that a degree of imperfection was desirable in these stimuli, or at least that participants did not demand perfect symmetry.

Another interesting finding was the absence of any significant evolution in the free-viewing group. It seems that bottom-up features that could attract gaze were insufficient to produce much systematic oculomotor behavior. This result supports the conclusion of Höfel and Jacobsen (2007), who claimed that preference formation requires intention and is not spontaneous. Participants in the free-viewing group could, in theory, have simply chosen to engage in a preference task even though they were not instructed to do so, and looked to the patterns they liked most. Although this may have happened sporadically, most participants did not show enough dedication to such a task to drive substantial genetic changes in the virtual population. This, again, tells us something interesting about preference formation: Our stimuli were *not aesthetically engaging* enough to elicit concerted evaluation during free viewing. We speculate that more interesting stimuli would lead to greater similarities between free-viewing and preference conditions. In other words, we cannot rule out the possibility that very different free-viewing results could emerge under other circumstances or with other stimuli. For example, if the starting population contained a noticeable degree of symmetry, and were designed so as to be highly aesthetically appealing, the participants could indeed engage in a spontaneous preference task and drive evolution. However, there is not evidence that this occurred in our free-viewing task.

We speculated that preferences might change with familiarity. Participants seemed to settle on their preference configurations less than halfway through the experiment. However, there was no evidence of further dynamic changes in after this. For example, we did not see any evidence that participants began to dislike symmetry once it became the norm. This conclusion is tentative because our experiments were still relatively short. The results cannot generalize to the extended dynamic interplay of familiarity and innovation that shapes fashion and consumer choice (Carbon, 2010) or aesthetics canons (Cutting, 2003) over decades. We can, however, claim that there was little evidence for comparable dynamics on a shorter timescale here.

Finally, we considered orientation of the reflection axis. Vertical symmetry is known to be more salient than other orientations when orientation is not predictable (Wenderoth, 1994). We predicted that vertical orientation could attract gaze, and there might be evolution toward vertical orientations in all the conditions to some extent. However, there was no systematic shift toward vertical orientations in any condition.

### Limitations and Considerations

The oculomotor genetic algorithm technique presented here is promising but exploratory. It is worth discussing four potential problems, before moving on to consider the advantages of the approach over alternatives.

First, we claim that participants did not demand perfect symmetry in the preference condition. What if the patterns were perceptually identical to perfect symmetry in later generations? That is, our participants liked perfect symmetry, and the level of symmetry achieved in the preference group *looked* perfect, so no further evolution occurred? In other words, do our results reflect a preference for imperfection, or merely the limits of visual symmetry discrimination? The latter can be countered by noting that patterns evolved to be significantly more symmetrical in the symmetry group than in the preference group. That means participants could have driven evolution toward a higher degree of symmetry if they found the patterns unsatisfying. This was not precluded by the limits of visual symmetry discrimination.

Second, we claim that the lack of a rebound to random in the preference group is theoretically interesting—it tells us that participants did not get bored with symmetry. However, variation in the virtual gene pool had reduced after the first few generations. Could evolution be stuck at the asymptote for trivial implementational reasons? We note that symmetry reached a *higher level* than this in the group of participants who were actively looking for symmetry. This suggests evolution was not stuck at the asymptote in the preference group. This also means that there was scope for a rebound toward random.

Third, interpretation of the free-viewing results is complicated. We *can* conclusively say that no substantial and consistent preferential viewing happened (unlike the preference condition), but we *cannot* say why. There are various plausible explanations. Perhaps preferential looking was masked by some other spontaneously chosen scanning strategy. For example, in the absence of instructions, participants (or just a significant proportion of participants) may have decided to look at each of the four patterns sequentially. Alternatively, participants could have engaged in spontaneous activities that increase local fixations *within* the stimulus rather *between* the four stimulus regions. Such oculomotor behavior may have dominated the influence of spontaneous preference on gaze, which would otherwise have driven evolution. Patterns that were more immediately salient might have produced very different results under free viewing. In future work, we intend to vary the phenotype of the starting population, to see if evolution-toward-symmetry can sometimes catch on under free-viewing conditions.

Fourth, we need to be cautious about equating the tendency to attract gaze with positive valence. There are many reasons people might fixate a pattern. Most obviously, there are low-level visual features that increase salience and attract exogenous attention. It is even plausible that stimuli with negative valence may attract gaze. When the algorithm assigned a high fitness score, this means the genes produced a pattern that was good at attracting the participants gaze: this does not necessarily mean the phenotype had *positive valence*. Researchers using the oculomotor GDEA should be mindful that, under free-viewing conditions, the GDEA technique only taps a *certain aspect* of aesthetic appeal, namely, the ability to attract gaze.

## Conclusions and Future Directions

One of the major methodological challenges for scientific aesthetics and consumer psychology is to find ways of measuring preference in ways that are not too artificial. Here, we explored the potential for the GDEA approach. We used preference for symmetry as a paradigm case, because it has previously been demonstrated in many species, using numerous different techniques. The approach allowed new insights into this well-trodden field. First, we learned that people do not always demand perfect symmetry. Second, we found no symmetry preference under free-viewing conditions, when there was no overt instruction to evaluate. Third, it was found that there was no change in preference for symmetry once it has become familiar.

The great advantage of the evolutionary algorithm approach is that it allows rapid quantification of fitness. Genotypes can be imagined as multidimensional space, with one gene on each dimension. We can then ask, what is the optimal position in this space? In nature, optimal simply means having the most offspring. However, in our simulation, “optimal,” means best at attracting and keeping the attention of a human observer. Here we did not actualize the full potential of the approach, because we only had three genes, and thus three dimensions, and only two of the genes were continuous. There were not a great number of possible genotypes that would result in perceptually distinct phenotypes. In future, the technique allows the addition of more genes to code factors that may influence preference. Inclusion of a color gene is an obvious next step, as people usually have a preference for blue-green over yellow-brown (Palmer & Schloss, 2010). Patterns could then be much richer, as local color would be more or less symmetrically positioned, and color preferences could interact with symmetry preferences in interesting nonlinear ways. If every possible combination was presented and rated explicitly, the addition of extra dimensions would result in an intolerable increase in the duration of the experiment. However, with the genetic algorithm approach, this problem is greatly reduced. This methodological advantage is important because the advancement of scientific aesthetics depends on being able to find preferences for multidimensional stimuli, rather than studying one dimension at a time, which is a serious limitation (Kubovy, 2000).

Indeed, as discussed by Holmes and Zanker (2012), aesthetic experience often relates to the gestalt, or whole, rather than the sum of isolated parts. The reductionist approach in empirical aesthetics is problematic because preference for the whole cannot usually be deduced by measuring preference for the parts one at a time (Arnheim, 1974). The polar opposite approach is to analyze people’s conscious introspections about what makes a final piece of art or design successful. However, introspective awareness of cognitive processes, such as those involved in aesthetic evaluation, may be far more limited than we generally assume (Nisbett & Wilson, 1977). The evolutionary algorithm approach is a promising new way to simultaneously avoid both these pitfalls.

The GDEA developed here could also be of use in consumer psychology, which is typically interested in preferences for particular package designs or graphics. This field faces all the same substantial challenges as scientific aesthetics. Again, there is the problem of finding an optimal point in a large multidimensional space and the problem of limited introspection. We think consumer psychologists should be maximally aware of these problems and could consider the GDEA as a possible way forward.
